# ‘The mercurial piece of the puzzle’: Understanding stigma and HIV/AIDS in South Africa

**DOI:** 10.1080/17290376.2015.1130644

**Published:** 2016-01-18

**Authors:** Leah Gilbert

**Affiliations:** ^a^ PhD, is Emeritus Professor of Health Sociology at the Department of Sociology, University of the Witwatersrand, Johannesburg, South Africa

**Keywords:** stigma, HIV/AIDS, South Africa, stigmatisation, VIH/SIDA, Afrique du Sud

## Abstract

Although stigma and its relationship to health and disease is not a new phenomenon, it has not been a major feature in the public discourse until the emergence of HIV. The range of negative responses associated with the epidemic placed stigma on the public agenda and drew attention to its complexity as a phenomenon and concept worthy of further investigation. Despite the consensus that stigma is one of the major contributors to the rapid spread of HIV and the frequent use of the term in the media and among people in the street, the exact meaning of ‘stigma’ remains ambiguous. The aim of this paper is to briefly re-visit some of the scholarly deliberations and further interrogate their relevance in explaining HIV-related stigma evidenced in South Africa. In conclusion a model is presented. Its usefulness – or explanatory potential – is that it attempts to provide a comprehensive framework that offers insights into the individual as well as the social/structural components of HIV-related stigma in a particular context. As such, it has the potential to provide more nuanced understandings as well as to alert us to knowledge-gaps in the process.

## Résumé

Bien que la stigmatisation liée à la santé et à la maladie ne soit pas un phénomène nouveau, les effets délétères de ce type de discrimination ne sont devenues des questions centrales dans le discours publique que lors des débuts de la lutte contre le VIH/sida. Les réactions négatives face à l’épidémie ont fait de la stigmatisation un phénomène d'actualité publique dont la complexité nécessite de plus amples réflexions. En dépit du fait que la stigmatisation et les discriminations liées au VIH/sida sont reconnues comme contribuant à la propagation rapide de la maladie et malgré l'utilisation ubiquitaire de ces termes dans la presse et bien que ceux-ci fassent partie du vocabulaire de la rue, la définition du terme ‘stigmatisation’ reste équivoque. Le but de cet article est de brièvement revoir certains des débats scientifiques sur le sujet afin de pouvoir avancer une étude sur leur pertinence dans le cadre du phénomène tel qu'il existe en Afrique du Sud. Un modèle sera proposé en conclusion. L'utilité de ce modèle – son potentiel explicatif – réside dans ce qu'il propose un cadre de référence détaillé sur les éléments aussi bien individuels que sociaux-structurels rattachés à la stigmatisation liée au VIH/sida dans un contexte particulier. Ce modèle propose un discernement plus nuancé de la situation existante en même temps qu'il met en évidence les lacunes qui subsistent.

## Introduction

Stigma and its relationship to health and disease is not a new phenomenon. It has been studied and examined in relation to various conditions such as epilepsy and mental health. However, it has not been a major feature in the public discourse until the emergence of HIV. The range of negative sentiments and responses associated with the epidemic placed ‘stigma’ on the public agenda and drew attention to its complexity as a phenomenon and concept worthy of further investigation by the academic community (Scambler 2009).

As early as 1987, Jonathan Mann, the former head of the World Health Organisation's global AIDS programme, highlighted what he termed the ‘third epidemic’, which he described as ‘the social, cultural, economic and political reaction to AIDS [which] is as central to the global challenge as AIDS itself’ (Mann 1987:1). Some thirteen years later, ‘stigma’ was again placed at the top of the list of ‘the five most pressing items on [the] agenda for the world community’, by Peter Piot, the Executive Director of UNAIDS, at the 10^th^ meeting of the agency's Programme Coordinating Board in 2000 (Parker & Aggleton 2003:14). The focus on stigma has steadily increased throughout the course of the epidemic, even becoming the focus of the World AIDS Campaign for the years 2002–2003 (Earnshaw & Chaudoir 2009). Yet despite its now prominent place in the public discourse as well as in scholarly literature, HIV-related stigma continues to be a serious public health concern.

There is widespread consensus that although stigma is not a new concept, it has acquired unique social significance in the context of HIV, particularly in South Africa (SA) (Gilbert & Walker 2010). Its impact on the epidemic and on people living with HIV/AIDS (PLWHA) has been widely discussed and analysed. Since the beginning of the epidemic, researchers have noted that the stigma associated with HIV is a barrier to prevention and treatment efforts, and, despite the worldwide attention, it continues to be a significant stumbling block in HIV programmes more than 30 years after the start of the epidemic. There is no doubt that HIV-related stigma compromises the well-being of people living with the disease. Many stigmatised individuals suffered discrimination, which leads to loss of employment and housing, estrangement from family and society, increased risk of physical violence and even murder (Walker, Reid & Cornell 2004). According to the latest Gap Report (UNAIDS 2014), which uses data from the People Living with HIV Stigma Index:

- On average, one in eight people living with HIV report being denied health services and one in nine are denied employment because of their HIV-positive status.
- An average of 6% reported experiencing physical assault because of their HIV status.

From a public health perspective, HIV-related stigma may fuel new infections, because it can deter people from getting tested (Kalichman, Simbayi, Jooste, Toefy, Cain, Cherry, *et al*. 2005), make them less likely to acknowledge their risk of infection and discourage those who are HIV-positive from discussing their HIV status with their sexual partners and others. It may also prevent them from accessing much-needed antiretroviral treatment (Mahajan, Sayles, Patel, Remien, Sawires, Ortiz, *et al*. 2008; Zuch & Lurie 2012).

However, despite the recognition that stigma as a phenomenon is one of the major contributors to the rapid spread of the epidemic (Rohleder, Swartz, Kalichman & Simbayi 2009), and the widespread use of the term in the media and colloquially, the exact meaning of ‘stigma’ remains ambiguous and hard to determine. Therefore, understanding its conceptual complexity and its embodiment in the reality of HIV and AIDS in SA presents a formidable challenge.

The quest to better understand stigma has preoccupied the academic community since the term was popularised by Goffman (1963), and attempts to conceptualise-and-re-conceptualise the notion of stigma have featured in many scholarly articles (Deacon, Stephney & Prosalendis 2005; Link & Phelan 2001, 2006). While these academic exercises provide a more nuanced understanding of the various dimensions of the concept, they further add to our confusion in trying to explain the complex manifestations of HIV-related stigma (Earnshaw & Chaudoir 2009).

The main aim of this article is to briefly review some of the scholarly deliberations with the view to examining their usefulness and relevance in explaining HIV-related stigma evidenced in SA.

## The development of the concept of ‘stigma’

Gilbert and Walker (2010) demonstrate some of the complex dimensions of HIV-related stigma as reflected in the literature. Goffman described stigma as ‘an attribute that is deeply discrediting within a particular social interaction’ (1963:3). His explanation of stigma focuses on the public's attitude towards a person who possesses an attribute that falls short of societal expectations. Goffman identifies three distinct types of stigma: stigmas of the body; stigmas of character; and stigmas associated with social collectivities, all of which he stresses are socially, culturally and historically variable. Juxtaposing HIV against Goffman's three potential attributes of stigma, it is evident that more than one attribute, and often all three, might apply at the same time – increasing the severity and complexity of stigma-related attitudes and behaviour in comparison to other conditions where only one of the attributes applies.

Although the concept of stigma has been applied to myriad social and medical circumstances, Goffman's ideas are a common thread in most of the studies and provide the theoretical underpinnings for much of the literature on stigma and stereotyping. According to Goffman and other scholars, diseases associated with the highest degree of stigma share common attributes: the disease is progressive and incurable, it is not well understood among the public and the symptoms cannot be concealed.

HIV fits the profile of a condition that carries a high level of stigmatisation. First, people infected with HIV are often blamed for their condition and many people believe HIV could be avoided if individuals made better moral decisions. Second, although AIDS is treatable, it is nevertheless a progressive, incurable disease, and more so in countries such as SA, where not all those in need of treatment have access to it. Third, HIV transmission is poorly understood by some people in the general population, causing them to feel threatened by the mere presence of the disease (Niehaus 2007). Finally, since HIV infection is asymptomatic, it can often be concealed. However, the symptoms of AIDS-related illnesses cannot: these symptoms may be considered repulsive, ugly and disruptive to social interaction.

Scambler (2004) makes a useful distinction between ‘felt’ and ‘enacted’ stigma. The former pertains to the fear of being discriminated against, while the latter refers to the actual cases of discrimination. Often, the fear of discrimination is more disruptive than the actual cases of enacted stigma. Stigma may be ‘enacted’ or ‘felt’ or both. Whereas enacted stigma denotes discrimination by others, felt stigma represents an internalised sense of shame and blame, respectively, and a frequently disruptive and sometimes disabling fear of being discriminated against (Cameron 2005).

Herek and Capitanio (1998) referred to ‘instrumental’ and ‘symbolic’ stigma: while ‘symbolic’ stigma is based on negative moral judgement and therefore is value-laden, ‘instrumental stigma’ is based on inflated fears and represents self-preservation from a perceived threat. This is an important distinction in the context of HIV because the mode of transmission via an everyday life event and incurability combine to make HIV understandably frightening.

Due to the variety of attempts to explain ‘stigma', there is confusion as to how various ‘types’ of stigma should be defined and how they relate to each other (Deacon & Stephney 2007; Deacon *et al*. 2005). Nevertheless, the negative effects of stigma on the lives of people diagnosed with chronic conditions, including those with HIV, have been well documented (Scambler 2009).

Kalichman, Simbayi, Cloete, Ginindza, Mthembu, Nkambule, *et al*. (2009) argue that there is evidence that AIDS stigmas can become ‘internalised’ and may therefore play a crucial role in the distress experienced by many PLWHA across cultures, as expressed by Cameron (2005) in his personal account of ‘felt’ and ‘internalised’ stigma.

HIV-related stigma is further complicated by other socially stigmatising characteristics of the groups most afflicted by the epidemic, leading to multiple stigma termed ‘layered stigma’, since it is often ‘layered’ over other forms of social inequalities in the areas of race, gender, class, sexual orientation and others (Deacon & Stephney 2007). As documented by UNAIDS (2014), people living with HIV who are members of key populations face a double stigma because of their sexual orientation, gender identity, drug use or engagement in sex work. Discrimination arising from HIV-related stigma is a response to the fears and prejudices of individuals and communities (Goudge, Ngoma, Manderson & Schneider 2009).

Manifestations of stigma vary according to time and place and are socially constructed (Scambler & Paoli 2008), but, typically, they follow core social structures or ‘fault lines of society’ such as class and gender (Castro & Farmer 2005; Eba 2008; Leclerc-Madlala, Simbayi & Cloete 2009). This is exacerbated by fear, ignorance, anxiety, denial, shame, taboo, racism, xenophobia, moral judgements and by misleading metaphors such as death, punishment, crime, war, horror and ‘otherness’ (Delius & Glaser 2005; Niehaus 2007; Posel 2005; Sontag 1989). In this context, Petros, Airhihenbuwa, Simbayi, Ramlagan and Brown (2006) found that the ‘othering’ of blame for HIV is central to social positioning, and is refracted through the multiple prisms of race, culture, homophobia and xenophobia.

In Goffman's tradition, much of the literature on stigma is on the ‘micro’ or individual level, which limits its explanatory power and creates the need to move beyond the narrow focus on the people affected by stigma to more ‘macro’ analyses. Expanding on Goffman's social interactionist definition of stigma, Link and Phelan (2001) conceptualise stigma as the co-occurrence of labelling, stereotyping, separating, status loss and discrimination. For this reason, Phillips, Benoit, Hallgrimsdottir and Vallance (2012) claim that their definition, with its more explicit focus on structural contexts, has fostered stigma research in two additional areas: the translation of stigmas into broader socio-cultural traditions and institutions, including social welfare policies, and the interaction of stigmas with other determinants of health advantage and disadvantage. Given this more recent structural focus, stigmas emerge as a wide-ranging social determinant of health, affecting not only identity formation and social interaction, but also access to a range of health and social welfare resources (Stuber, Meyer & Link 2008). This line of thinking introduces an additional, most crucial, component of stigma: the exercise of power, which clearly points to the fact that wide social differentials are a pre-requisite for the instigation of stigma. Its significance in further understanding stigma and its relation to health in particular are clearly indicated by the fact that a recent issue of *Social Science & Medicine* was devoted in its entirety to ‘Structural Stigma and Population Health’, where the concept was further interrogated (Hatzenbuehler & Link 2014).

Similar ideas have been articulated by Parker and Aggleton, who adamantly reject the individualism underlying conventional approaches to stigma and its alleviation. Instead they insist that

stigma and stigmatisation function, quite literally, at the point of intersection between *culture, power and difference* – and it is only by exploring these different categories that it becomes possible to understand stigma and stigmatisation not merely as an isolated phenomenon, or expression of individual attitudes or of cultural values, but as central to the constitution of the prevailing social order. (2003:17)

They further argue that it is especially important to think of stigma as a social and cultural phenomenon linked to actions of whole groups of people particularly in the developing world, where bonds and allegiances to families, village, neighbourhood and community abound. Mahajan *et al*. maintain that

theorizing stigma in this way also highlights the necessity of power – social, economic, or political power – to enable a community to move from individual-level perceptions to collectively identify an undesirable difference/attribute, construct stereotypes and, ultimately, to act on the negative stereotype by discriminating against the stigmatised. (2008:70)

These explanatory notions are taken further in Link and Phelan's development of the term of ‘stigma-power’ as a resource that refers to ‘instances in which stigma processes achieve the aims of stigmatisers with respect to exploitation, management, control or exclusion of others’ (2014:24). In their analysis, they draw on Bourdieu's (1987, 1990 cited in Link & Phelan 2014) concepts of symbolic power and misrecognition and argue that the many stigma processes serve the interests of stigmatisers in subtle ways that are difficult to recognise. Although they focus on the role of stigma-power in mental illness, by drawing attention to the structural factors, their suggestions have the potential to shed more light on understanding HIV-related stigma and advance the thinking about ways to reduce its impact.

Although most of the literature on stigma has focused on those who experience stigma directly, Goffman (1963) suggested that stigmas affect not only the individuals bearing them, but also those who are closely associated with stigmatised individuals and groups. Phillips *et al*. (2012) point out that despite this initial foray into the concept of ‘courtesy stigma’ (also called ‘stigma-by-association’ or ‘associative stigma’), relatively few studies have attempted to study its impact on the everyday lives of those who support stigmatised people. However, from the material available in the studies to date, it emerges as a prevalent phenomenon that requires further research and consideration (Ogunmefun, Gilbert & Schatz 2011).

## Examining the evidence

The broad spectrum and variability of the concept of stigma render it hard to evaluate and ‘measure’ (Earnshaw & Chaudoir 2009). This, as neatly stated by Abrahams and Jewkes (2012:2), ‘makes the assessment and comparison of the huge body of research that emerged globally and in South Africa difficult’. Bearing in mind this problematisation of the concept, I would like to briefly examine the ‘evidence’ based on some of these studies.^1^ Consistent with the aims of this paper, this will be done in order to highlight the points made earlier and to demonstrate the challenges in understanding and explaining stigma as it has manifested so far.

Based on empirical evidence, Simbayi, Kalichman, Strebel, Cloete, Henda and Mqeketo (2007) claim that HIV is perhaps the most stigmatised medical condition in the world. I agree, and would add that the nature and magnitude of ‘stigma’ has a historical as well as geographical dimension: it has been changing since the onset of the epidemic and has manifested in various forms in different countries, mainly along the fault lines of ‘developed’ versus ‘developing’, and linked to access to health care and treatment, which highlights the significance of structural stigma, as mentioned earlier. A full exploration of these crucial dimensions is, however, beyond the scope of this paper.

The evidence that dominated the academic literature as well as the global ‘lay’ media in the early days of the epidemic was that of ‘enacted stigma’ or actual discrimination in the form of denial of health care, job dismissals and rejection by family members, as systematically reported by Panos Dossier (1990). Similar cases occurred in SA, such as the much-publicised refusal to admit Nkosi Johnson as a pupil to a primary school (Simon 1997). Or that of Gugu Dlamini, who was murdered by members of her community a month after disclosing her HIV status on a provincial radio station (Raubenheimer 1999) as well as other cases covered by local and international media (McGeary 2001; Paton 1997; Seeger 1998). These cases are clear displays of both ‘symbolic’ and ‘instrumental’ stigma as discussed by Herek and Capitanio (1998).

In an attempt to understand – and ameliorate – these troubling effects of the ‘third epidemic’, many scholars predicted that with the spread of the epidemic and the increased exposure to PLWHA that is likely to follow, these extreme manifestations would most likely decline, but at the same time they recommended legislative efforts and public health programmes to counteract these harmful effects (Goldin 1994; Malcolm, Aggleton, Bronfman, Galvo, Mane & Verral 1998).

Indeed, this most extreme negative scenario as described above (such as refusal of admission or murder) has slowly been altering as the epidemic has unfolded. This was accompanied by changes in existing – and the introduction of new – anti-discriminatory legislation and the initiation of programmes and awareness campaigns to reduce these negative aspects of the epidemic and the intense stigma associated with it. At the same time, the medical establishment has unsuccessfully tried to find a vaccine and/or cure for this devastating epidemic. Studies at that time focused mainly on the stigmatising experiences of PLWHA (Campbell, Foulis, Maimane & Sibiya 2005; Green 1995; Reid & Walker 2003; Richter 2001) as well as people's perceptions of and attitudes towards PLWHA in an attempt to assess levels of stigma among the general population (Shisana & Simbayi 2002). The main aim, however, of these studies was not to explain or to interrogate the conceptual complexity related to the existence of HIV-related stigma.

The turning point as far as the evolution of the epidemic is concerned was the successful introduction of effective antiretroviral therapy (ART). This transformed HIV and AIDS from an acute/fatal disease into a long-term chronic condition, giving PLWHA renewed hope and often a ‘second life’ (Gilbert & Walker 2010). However, at the same time this shifted the attention from the ‘social–cultural’ aspects of the epidemic to the ‘bio-medical’ forces shaping its development (Mykhalovskiy & Rosengarten 2009). As foreseen by many, this occurrence was also associated with a reduction of overt manifestations of stigma, mostly in the developed world in countries that have universal access to ART (Genberg, Hlavka, Konda, Maman, Chariyalertsak, Chingono, *et al*. 2009).

In SA, this process has not been as smooth because of former president Thabo Mbeki's denialist approach, followed by the government's reluctance to introduce ART in its public health facilities (Nattrass 2007). This once again emphasised the gap between the minority (∼20%) who have access to private health care and the majority of the population (∼80%) who rely on public healthcare services and were therefore denied access to ART – an unfortunate turn of events that highlights the forces of social inequalities at play in the context of HIV stigma as suggested by Parker and Aggleton (2003) and further, more recently, theorised by Phelan, Lucas, Ridgeway and Taylor (2014).^2^

Under immense public pressure, a universal ART roll-out in the public service in SA was finally announced in 2004, and with it came the hope that a decline in stigma would follow. Since one of the main reasons for the high levels of HIV-related stigma in SA was its association with death (Niehaus 2007), it has been assumed that with the growing access to ART and the increased number of PLWHA – not dying of it – levels of stigma would go down. However, the evidence tells a different story, as will now be outlined.

It is useful at this stage to take cognisance of Herek's (2002) warning that while disease stigma historically decreases as the disease is better understood and as treatment becomes available, this appears not always to be the case with regard to HIV. He suggests that the general public remains poorly informed about HIV, and that the scientific information about HIV is often not trusted, particularly if political figures openly question the science around antiretrovirals, as was the case in SA. This gives credence to Link and Phelan's (2014) notion of stigma-power as discussed earlier.

Maughan-Brown (2010) set out to explore the changing nature of HIV-related stigma and the potential determinants of these changes following the ART roll-out. Using longitudinal data from two surveys conducted in 2003 and 2006 among a cohort of young adults in Cape Town, SA, he reported that HIV-associated stigma had increased and, more specifically, that knowing someone who had died of HIV-related diseases increased both instrumental and symbolic stigma. Increased personal contact with PLWHA was not significantly associated with changes in stigma. Indeed, as attested by the study's title, ‘stigma rises despite antiretroviral roll-out’ (Maughan-Brown 2010:368).

In addition to the evidence above, studies have determined that the courtesy stigma produced similar results. Ogunmefun *et al*. (2011) found that older female caregivers in rural SA had experienced substantial secondary/courtesy stigma because they were looking after family members with HIV. The authors classify the types of secondary stigma experienced by the carers. These included physical stigma in the form of isolation and separation from family members; social stigma in the form of voyeurism and social isolation; and verbal stigma in the form of being gossiped about, finger-pointing and jeering at them.

Similarly, in a study of perceived stigma among patients receiving ART in KwaZulu-Natal, Peltzer and Ramlagan (2011) found that despite a decrease in stigma seen in their study – suggested to be due to ART – the level of stigma and discrimination remained high. For this reason, they recommended that stigma-reduction interventions were urgently needed in this population.

As part of a specific effort to reduce the high HIV-related maternal mortality in SA, Turan, Nyblade and Monfiston (2012) examined how stigma acts as a barrier at each step in the complex series of interventions that woman and infants must complete for successful Prevention of Mother-to-Child Transmission (PMTCT). The Health Policy Project team reviewed the existing literature to examine the current evidence on stigma and discrimination and their negative impacts on PMTCT and family health. In their extensive report based on both quantitative and qualitative data from low-resource settings worldwide, they reveal the negative effects of fears and experiences of HIV-related stigma and discrimination that begin with the initial use of services during pregnancy and continue to affect PMTCT and maternity service use throughout pregnancy, birth, and the postnatal period. This confirmed that stigma and discrimination are key barriers to achieving global goals for maternal health and the elimination of new child HIV infections in 2012 despite the availability of effective medical solutions.

Maman, Abler, Parker, Lane, Chirowodza, Ntogwisangu, *et al*. (2009) compared HIV stigma in five international sites with a view to examining the influence of care-and-treatment resources in high-prevalence settings and concluded that the family, access to antiretrovirals and other resources, protected against HIV-stigma and discrimination. They also found that social inequalities in the form of variation in the availability of health and socioeconomic resources help explain differences in HIV stigma across the settings in their study. However, they argue that despite the fact that increasing access to treatment and care resources may function to lower HIV stigma, providing services is not enough. Therefore, they maintain that ‘We need effective strategies to reduce HIV-stigma as treatment and care resources are scaled up in the settings that are most heavily impacted by the HIV epidemic’ (2009:2271).

In another international study, Genberg *et al*. (2009) conducted a comparison of HIV-related stigma in four countries, and revealed negative attitudes and perceived acts of discrimination towards PLWHA. More negative attitudes were found in sites with the lowest HIV prevalence (i.e., Tanzania and Thailand) and more perceived discrimination against PLWHA was found in sites with the lowest ART coverage (i.e., Tanzania and Zimbabwe). Based on their findings, they conclude that ‘Programs that promote widespread HIV testing and discussion of HIV/AIDS, as well as education regarding universal access to ARVs, may reduce HIV/AIDS-related stigma and discrimination’ (2009:2279).

Indeed, this is a sensible component in stigma-reduction public health interventions, as revealed in a study by Mall and colleagues. They used data from two consecutive community-based, cross-sectional surveys, performed four years apart, to describe the changes in stigma, HIV-knowledge and voluntary conseling and testing access over time in a community with high HIV prevalence. Despite its limitations, this study demonstrated ‘that levels of HIV-associated-stigma can be reduced over time in a community burdened with a high HIV-prevalence, and this reduction is associated with an increase in reported HIV testing’ (2013:200).

Phillips *et al*. (2012) draw attention to ‘courtesy stigma’ as a hidden health concern among frontline service providers to sex workers. In light of their findings, they maintain that assessing the relative impact of primary stigma and courtesy stigma versus other determinants of health is a complex issue. Therefore, they further argue that the insights of stigma scholars that highlight the interconnectedness of various axes of marginalisation, as well as the interaction between social determinants of health, should be seriously considered in this context.

## Discussion

Given the negative effects of the ‘third epidemic’, scientists and activists have put a vast amount of effort into reducing its impact and educating the public that ‘stigma’ and discrimination are socially objectionable (Sengupta, Banks, Jonas, Miles & Smith 2011; Steinberg 2008). Anti-discriminatory legislation was put in place to protect the rights of PLWHA and ‘anti-stigma’ educational campaigns mounted – putting ‘stigma’ as a concept on the public agenda. The educational initiatives introduced ‘stigma’ into the public's discourse and emphasised its offensiveness and undesirability. The end result today is that most people are aware of the negative attributes of the concept and its social unacceptability, and therefore – I would venture to suggest – there has been a reported decrease in ‘stigma’, particularly as evidenced by results from nationwide surveys (Shisana, Rehle, Simbayi, Zuma, Jooste, Pillay-van-Wyk, *et al*. 2009). However, as pointed out in this article, the reality on the ground is that despite the introduction of ART, stigma continues unabated, and is often disguised in different, more elusive forms (Abrahams & Jewkes 2012; Naidoo, Uys, Greeff, Holzemer, Makoae, Dlamini, *et al*. 2007). Further evidence to substantiate the on-going existence of stigma is provided in the latest Gap Report (UNAIDS 2014).

There is no dispute that growing awareness that it is unacceptable to discriminate against people with HIV is an important step forward, but it is not quite the same thing as a real reduction in stigmatising attitudes and behaviours. For these reasons, 12 years later I concur with Stein (2003), who argued that HIV stigma has not in fact diminished but has, rather, become another ‘dirty secret’. This further problematises its ‘measurement’ and raises the question about ‘how to measure a hidden truth?’

Although this question has not yet been fully answered, the need to ‘measure’ stigma is reflected in the literature (Kalichman *et al*. 2005, 2009) and has produced a useful index to gauge levels of discrimination felt by people living with HIV that has been used in over 50 countries since its inception in 2008 (The People Living with HIV Stigma Index 2014).

Note should be taken, however, that my stance does not suggest that HIV education is not an important component in efforts to decrease stigma, but, rather, that access to information on its own is insufficient to eliminate existing myths and beliefs regarding transmission, or, for that matter, to eradicate stigma as evidenced by many of the studies cited here.

## Conclusion

In this article, I set out to highlight the conceptual complexity of stigma through the questions that have been central in the academic literature. The main aim of this article has been to briefly re-visit some of the scholarly deliberations and further interrogate their relevance in explaining HIV-related stigma evidenced in SA. Although it provides more nuanced understandings of the concept, most of the literature reviewed adds a level of complexity that requires further investigation and renders comprehension of stigma more problematic. Due to the focused nature of this article, only brief mention was made of the structural forces related to stigma in SA; however, cognisance of their complex impact needs to be taken. This has also been argued by Deacon and Stephney when they claimed that ‘the process linking stigma and disadvantage is much more complex, and the solutions we must seek to both problems are also much more complex’ (2007:6).

For this reason, I would like to put forward the argument that the existence of such an abundance of scholarly articles on the concept and its definitions is testament to the fact that they fall short in providing a full explanation for the various manifestations of stigma and its relationship with other social forces. In addition, the explosion of studies looking at ‘measuring’ stigma and reducing its impact is evidence that stigma exists out there and continues to be a threat to the successful implementation of public health programmes.

Indeed, given its varied dimensions and complex nature, ‘stigma’ remains a hotly debated concept among scholars (Tal 2012). This is so to the extent that in an editorial, ‘Stigma Research and Action’, Tal asks the question whether it is time to retire the term stigma, since ‘not everyone is comfortable with the term [stigma] and its connotations’ (2012:49), and a series of articles from diverse countries in the world comment on and address this debate from different perspectives (Holley, Stromwall & Bashor 2012).

Based on the evidence and debates introduced here, there is no doubt that understanding the concept in the context of HIV in SA presents a serious challenge to sociologists and other social scientists. However, the challenge for public health is even greater, ‘since stigma remains the mercurial piece of the HIV puzzle – impossible to comprehend and tackle programmatically in the way we have mastered clinical management of HIV through understanding its virology’ (Crabtree 2012:7).

Since stigma has been identified as one of the barriers to the successful prevention of new HIV infection, it needs be tackled in a manner similar to general health promotion efforts. This is discussed by Gilbert, who claims that

There is no doubt that if the ultimate goal is to affect the ‘social’ in the epidemic, public health efforts need to focus on integrated and relevant ‘structural interventions’ along the continuum of the disease trajectory that correspond with the main drivers of the epidemic.^3^ (2012:74)

**Fig. 1.**
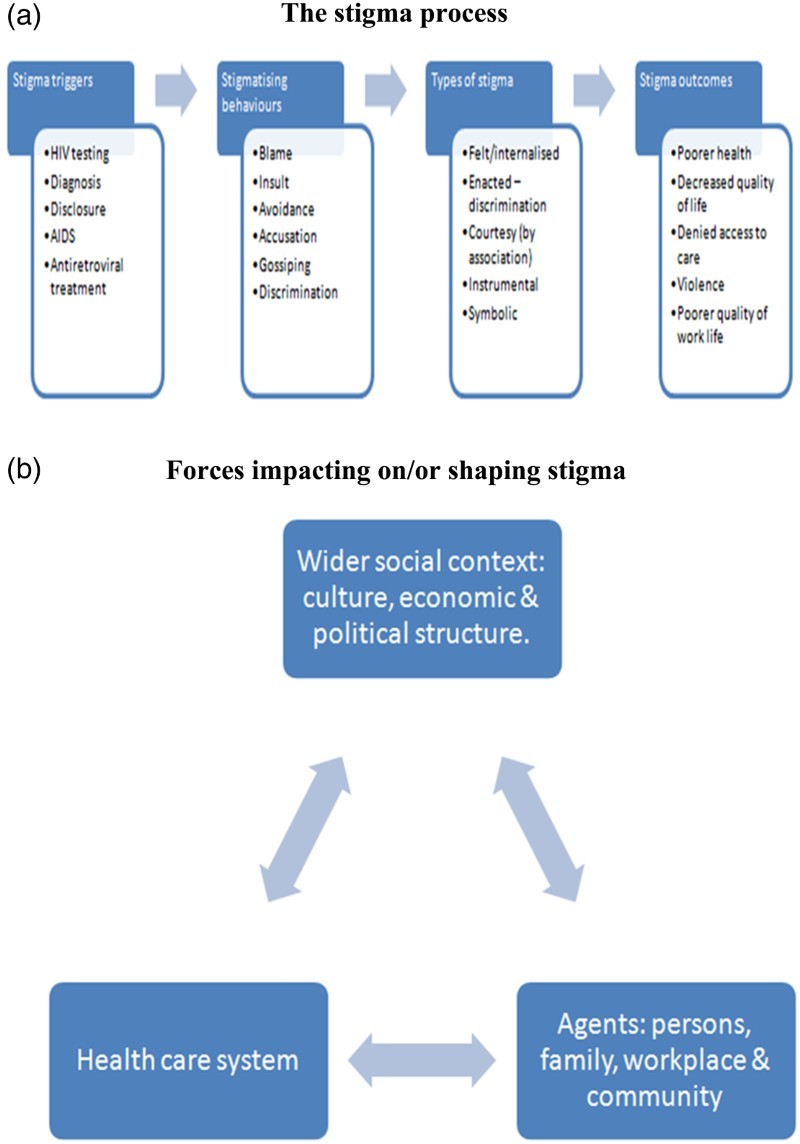
(a) The stigma process. (b) Forces impacting on/or shaping stigma.

It seems that the various attempts to conceptualise stigma and to better understand its impact on health have, so far, not provided all the answers. Based on a study in five African countries, Holzemer, Uys, Makoae, Stewart, Phetlhu and Dlamini developed a conceptual model delineating contexts and processes of HIV-stigma as reported by PLWHA (2007:546). Using this model as an inspiration, I would like to propose a more comprehensive model that combines the individual components of stigma as a process (Fig. 1(a)) as well as the wider framework of forces shaping the manifestations of stigma (Fig. 1(b)) based on the social-ecological model (Gilbert 2012).^4^

It is important to state that a ‘model’ remains a simplified representation of a more complex situation. However, elucidating the various components of the model has the potential to provide health professionals as well as activists with more specific issues to focus on. As presented in the first part of the model (Fig. 1(a)), the focus is on the ‘stigma process’ as it occurs on the individual level. It includes the triggers to the stigma such as HIV testing, diagnosis, disclosure, AIDS and ART. This is followed by a range of stigmatising behaviours such as blame, insult, avoidance, accusation, gossiping and discrimination. There are different types of stigma experienced on this level such as ‘internalised/felt’, enacted, courtesy, instrumental and symbolic stigma followed by stigma outcomes of poorer health, decreased quality of life, and denied access to care and violence.

The above reality of the ‘stigma process’ as analysed in this paper is not taking place in a social vacuum. For this reason, as discussed earlier, there is a need to take cognisance of the wider framework of the social context in which the stigma is occurring. This is provided in the second part of the model (Fig. 1(b)) that is depicting, on the one hand, the potential interrelationship between culture, economics, political structure, health care system, family, workplace and the community, and on the other hand, their probable impact on the ‘stigma process’ and the likelihood that these wider factors play a significant role in shaping it.

This might be helpful in coming to a better understanding of the fluid dynamics of stigma as a process and its complex embodiment in the context of HIV in SA. Its usefulness – or explanatory potential – is that it attempts to provide a comprehensive framework that offers insights into the individual as well as the social/structural components of HIV-related stigma in a particular context. As such, it has the potential to provide more nuanced understandings as well as to alert us to knowledge-gaps in the process as well as their practical implications.

Fig 1(a) and 1(b) makes it clear that stigma occurs on various levels.

Therefore, it seems that such a comprehensive model of stigma might be useful in programmes aimed at reducing levels of stigma by directing efforts towards the various components of stigma on the individual, community and societal level. But this can be done only with a better understanding of the concept, its manifestations, assessment and measurement. The challenge, therefore, is still out there.

## Notes

1. Since the aim of this paper is not to provide a comprehensive literature review on stigma-related- studies but rather offer a more nuanced insight, the studies cited here do not represent an exhaustive list.

2. A full discussion on the impact of social inequalities such as gender, race and class on HIV is beyond the scope of this paper. For further references, see Gilbert & Walker, 2002; Leclerc-Madlala et al., 2009; Gilbert & Selikow, 2011.

3. This article provides a comprehensive analysis of the significance of structural interventions in public health, which is of relevance to HIV-related stigma as well.

4. For additional background on this model, see Gilbert, 2012.
